# Risky Behaviors and Health-Promoting Behaviors in Young Adults: An Epidemiological Study

**Published:** 2019-10

**Authors:** Leila Jahangard, Helen Behmanesh, Mohammad Ahmadpanah, Seyedeh Mahsa Poormoosavi, Alireza Solitanian, Mohammad Highighi

**Affiliations:** 1 Research Center for Behavioral Disorders and Substance Abuse, Hamadan University of Medical Sciences, Hamadan, Iran.; 2 Department of Histology, School of Medicine, Dezful University of Medical Sciences, Dezful, Iran.; 3 Department of Biostatistics, School of Public Health, Hamadan University of Medical Sciences, Hamadan, Iran.

**Keywords:** *Adult*, *Behavior*, *Healthy*, *Risky*

## Abstract

**Objective:** Health behaviors are defined as activities that affect either health status or disease risk. They can be divided into 2 categories: risky behaviors and health promoting behaviors. The growing body of evidence indicates that unhealthy behaviors often cluster in young individuals. Patterns of health-related behaviors are significantly different among countries and even among various regions of a certain country.

**Method** **:** The present study was conducted to assess the youths’ patterns of health attitude, health-related behaviors, and their mental and physical wellbeing. In this cross-sectional study, 800 university undergraduate students were selected using multistage cluster sampling method. Standard questionnaires were filled by students.

**Results: **About 13.3% of students smoked regularly and 14.3% reported at least one occasion of drinking, and heavy drinking was quite prevalent. Of the students, 95% reported regular physical activity and exercise. Eating habits were not healthy among the majority of students, as there was a high consumption of fast food and salt, and only 23.9% had normal body weight. Self-care behaviors were not prevalent among the students (3.2% breast self-exam and 8.5% testicular self-examination).

**Conclusion: **Many factors may affect positive and negative heath behaviors, including knowledge, beliefs and attitudes, legal constrains, social context, and economic status. However, lower health literacy leads to more negative health behaviors.

Chronic non-communicable diseases (NCDs) have an increasing burden and are a major public health concern worldwide. They are major causes of morbidity and mortality but are often neglected in health system programs (1). The most important modifiable risk factors of the main chronic NCDs include unhealthy diet, physical inactivity, and tobacco use. Up to 80% of incident cases of cardiovascular disease and type 2 diabetes are attributable to these 3 factors (2). In addition to the burden of disease attributable to an individual behavioral risk factor, the growing body of evidence also indicates that unhealthy behaviors often cluster in young individuals (3) and that their combination bear higher risks for chronic diseases than the sum of their individual independent effect (4). 

WHO defines health risk as “a factor that raises the probability of adverse health outcomes” (5). People from low-income countries are particularly at risk for poverty-related risk factors, including poor nutrition, indoor air pollution, and lack of access to proper sanitation. However, the pattern of mortality and morbidity increasingly becomes convergent in both low- and high-income countries and non-communicable diseases such as cancers, cardiovascular diseases, and diabetes impact both rich and poor countries (6). Developing countries are increasingly being faced by NCDs around the globe, while still being challenged with traditional health problems (7). 

Health behaviors are defined as activities that affect either health status or disease risk and can be divided into 2 categories: risky behaviors and health-promoting behaviors. The former are activities that while repeated contribute to the development of chronic diseases; and the latter are precursors of health promotion and maintenance, including wide range of activities (regular physical activity, healthy eating, using sunscreens, fastening seatbelt, etc.) (8). 

Increasing risk awareness is known to be the essential factor in choosing a healthy life style. For example, the rate of smoking that was reduced as the role of cigarette smoking in lung cancer has been publicized in the past 20 years. However, knowledge of the health risks is not always a sufficient condition to change behavior. Sugar consumption is relatively high despite awareness of dental disease or diabetes risk (9, 10). 

Patterns of health-related behaviors are significantly different among countries and even among various regions of a certain country. Iran has a relatively high percentage of young population. The number of people aged 20-34 years is estimated to be more than 23 million, constituting 30% of the country’s population (11). Young people are the future citizens, parents, and workers of the country, especially those with higher education will have an important role in economic and social well-being of the nation, so their attitudes and health habits deserve special attention. There are few studies about the prevalence and patterns of risky behaviors among young Iranian population; however, studies that focus on health-promoting behaviors are scare. 

The present study was conducted to assess the youths’ patterns of health attitude, health-related behaviors, and their mental and general physical well-being. 

## Materials and Methods

***Study Design, Participants, and Sampling***

The survey was conducted in cities of Hamadan and Dezful and the study participants were university undergraduate students aged 18-44 years. Inclusion criteria were being an undergraduate student, being able to participate in the study, and willingness for participation. There were no specific exclusion criteria.

In this cross sectional study, 800 university undergraduate students were selected using multistage cluster sampling method. Information about the study purpose and method were provided to the participants, and those students who agreed to participate entered the study. Informed consent was obtained from all participants.

***Measures***

***General and Sociodemographic Information***

Personal information, including age, gender, marital status, field of study, number of years since entering university and current residence and living conditions, were collected using a questionnaire.

***International Health and Behavior Study (IHBS) Questionnaire***

IHBS questionnaire is a self-report questionnaire developed by Steptoe et al to evaluate health-related behavior, risk awareness, and associated attitudes in university students (12, 13). This questionnaire includes 6 sections: (1) measures of personal health behaviors, including dietary choice, physical activity, smoking, and alcohol consumption, sleep patterns, behaviors related to weight control, driving, dental care, and breast and testicle self-examination; (2) rating the importance of health behaviors; (3) assessing the awareness of risks associated with a range of personal health behaviors; (4) standard measures of sense of control, self-rated health, and life satisfaction, socioeconomic background, and religion; (5) scales on locus of control; and (6) the short-form of Beck Depression Inventory. The original questionnaire was in English and was translated into Persian and then back-translated to English by an independent translator who had no knowledge of the questionnaire. Emphasis in the back-translation was mainly on conceptual equivalence. Discrepancies were discussed until the satisfactory version was reached.

***Breslau's 7-Item Screening Test for Posttraumatic Stress Disorder (PTSD)***

Breslau's 7-item screening test for PTSD was developed by Breslau et al, with a sensitivity of 80%, specificity of 97%, positive predictive value of 71%, and negative predictive value of 98% (14). 

***The Alcohol Use Disorders Identification Test-Consumption (AUDIT-C)***

The AUDIT-C is a brief screening tool for alcohol use disorders and includes 3 questions, each with the score of 0–4, with scores greater than or equal to 4 in men and greater than or equal to 3 in women indicating risky drinking behavior, alcohol abuse, and dependence (15). 

***Anthropometric Measurements***

Anthropometric measurements were assessed by trained researchers using standardized techniques. Height was measured with accuracy of 0.1 centimeters and weight with a digital scale with accuracy of 0.01 kilogram. The scale was first calibrated using a standard weight and rechecked daily. Body mass index (BMI) was calculated using the formula of body weight (Kg)/height (m2). 

***Statistical Analysis***

Frequencies and proportions were calculated in number and percent using chi-square test. Multivariate logistic regression was used to assess the associations between variables. Data were analyzed using the statistical package SPSS 22.0, and significance level was set at p < 0.05.

***Ethical issues***

The study protocol was approved by the ethical committee of Hamadan University of Medical Sciences. Information about the objective of the study was presented to the participants before the application of forms, and informed consent was obtained. Also, data were collected anonymously and confidentiality principle was observed during the study***. ***

## Results

A total of 800 students participated in the survey, of whom 400 were female and 400 were male. The mean age of the participants was 22.03±3.36 yrs. The sociodemographic distribution of the participants is summarized in Table 1. 

In terms of life satisfaction, 40.2% of the students described themselves very satisfied, 25.1% satisfied, 26.9% unsatisfied, and 7.7% very unsatisfied (Table 2). Overall, females were more satisfied than males.

***Substance Use***

Tobacco use: Of the participants, 13.3% were current regular smokers (smoking almost daily) and 36.1% were occasional or experimental smokers. Smoking tobacco was more prevalent among males than females (OR= 1.63; P < 0.05). Smokers were more likely than nonsmokers to live in the dormitory, but this association was not significant (OR = 1.30; P > 0.05), and they reported significantly less overall health condition, educational achievement, and life satisfaction. Family financial status and ethnicity were not associated with tobacco use (Table 3). 

Alcohol Use: Of the participants, 14.3% reported at least one occasion of drinking during the last 12 months, and of them 2% were regular drinkers, 1.8% intermittent, and 3.4% occasional drinkers. The mean units of alcohol used in one occasion was 2.3 and almost all (96.4%) students who consumed alcohol at least once during the past 12 months reported at least one occasion of heavy drinking (more than 5 servings in males and more than 4 servings in females). More males reported at least one drinking in the past 12 months compared to females (p = 0.02). 

Prescription Drug Misuse: Of the students, 41.5% had at least one misused prescription drugs the past 12 months. Drug misuse prevalence was not significantly different in males and females (p = 0.44). 

***Physical Activity***

Of the participants, 63.2% had heavy physical activity, including power lifting, aerobics, or rapid spinning, at least once a week. Rates for moderate and light physical activity were 65.6% and 86.5%, respectively. In total, 95% of students reported regular physical activity. Rate of engaging in heavy physical activity reduced in students with higher educational level (p = 0.038), but this pattern was reverse for light physical activity. 

***Eating Habits***

Evaluations of consumption or skipping particular meals with respect to age and gender revealed that 52.3% of students had breakfast regularly regardless of gender and educational level and 47.9% took meals regularly; regular eating pattern was more frequent among male students (p = 0.01). Moreover, 84.3% of the students were taking snacks between meals. 

Also, 91.7% of the participants had at least one serving of fruit and vegetable daily, and almost all of them (99.9%) had fast food at least once in the previous week, and 61.3% usually added salt to their foods. 

Only 14.9% of the students perceived their body weight as normal, but only 23.9% had normal BMI, and 58.4% of the participants considered regimens for weight loss and tried to avoid high fat foods. 

***Risk-Taking Behaviors, Potentially Traumatic Events, and PTSD Symptoms***

Half of the students who had a sexual relationship, reported that their first sexual relationship occurred at the age of 18 and one-quarter were under the age of 15 at the time of their first sexual relationship. Also, more than half of them (52.2%) reported that their last sexual contact was unprotected.

Two thirds of the students (64.7%) always fastened seatbelts while driving or sitting in the front seat of the car. Among students who drove, only 13.2% stated that they never speed, although 59.9% considered it important. Also, 27.8% reported driving under the influence of alcohol and 15.9% had been in a vehicle whose driver was under the influence at least once during the previous year. Moreover, 31.8% of the students who rode motorcycles always wore helmets, and 51.1% reported that they had been involved in motor vehicle accident, and 6.9% had injuries that needed medical attention. 

Of all participants, 11.8% reported being involved in physical fights at least once in the previous year, and 13.1% had injuries other than motor vehicle accidents. 

Furthermore, 132 students (17.9%) reported being physically abused by their partner and 47 (5.9%) reported being forced for sexual relationship. Using Breslau's 7-item screening test for PTSD, 40.9% of the students were screened positive for PTSD symptoms, which was significantly higher among females. 

***Self-Care Behaviors***

Most of the female students (75.8%) knew how to do breast self-exam and half of them (50.4%) considered it to be important, but only 3.2% did it regularly (10 times a year or more). Of the female participants, 37.9% knew the importance of having regular Pap smear tests, but 93% had never done it. Among male students, only 9.4% were aware of the importance of testicular self-examination and as few as 8.5% did it regularly. 

**Table 1:** Sociodemographic Status of the Participants

		**Number = 800**	**Percentage**
	Age, year (M±SD)	22.03±3.36	
Gender			
	Male	400	50
	Female	400	50
Marital status			
	Single	88	11
	Married	699	87.4
	Other (nonresponse, etc.)	13	1.6
Family economic status			
	Wealthy	16	2
	High economic status	292	36.5
	Middle-income	454	56.8
	Low-income	23	2.9
Residence			
	Dormitory	629	78.6
	Private	154	19.2
	Other	17	2.2

**Table 2:** Descriptive and Inferential Statistical Indices of Life Satisfaction, Separately for Gender

	**Male (n = 400)**	**Female (n = 400)**	**P value (Chi square ** **test)**
Life satisfaction	N(% )	N (%)	0.001
Very satisfied	180 (45)	142 (35.5)	
Satisfied	81 (20.2)	120 (30.0)	
Unsatisfied	120(30.0)	95 (23.7)	
Very unsatisfied	19 (4.8)	43 (10.8)	

**Table 3:** Descriptive and Inferential Statistical Indices of Tobacco Use, Separately for Gender, Residence and Health Condition

		**Nonsmoking (n = 585)**	**Smoking (n = 215)**	**OR (95% CI)**	**P value**
**Gender**					
	Female	314	86	1.00	
	Male	271	129	1.73 (1.26, 2.38)	0.001
**Residence**					
	Non-dormitory	142	29	1.00	
	Dormitory	497	132	1.30 (0.83, 2.02)	0.245
**Health condition**					
	Satisfied	393	150	1.00	0.002
	Unsatisfied	212	65	167(1.18,2.34)	

## Discussion

The findings of this study found the regular smoking rate to be 13.3% and occasional and experimental smoking up to 36.1%. Previous studies among university students in Iran found the smoking rate of 6.2% and 31.5% (16). Furthermore, the findings showed that the rate of smoking among students was different from those reported in previous studies worldwide. A review which investigated smoking behavior among undergraduate students in the US, reported the rate of 40.2% for smokers and 28.1% for experimental smokers. In other countries, the prevalence of smoking among students was reported from 13% in Hong Kong (17) to 43% in Turkey (18). Relatively lower rates of smoking may reflect that the adolescents who smoke are less likely to enter the university due to concurrent behavioral problems resulting in educational failure and social obstacles. Unlike the above studies, gender difference in the smoking rate was significant in the present study. The different pattern of smoking between the two genders is similar to the pattern described few decades earlier in the developed countries (19). Hagen et al explained lower rates of smoking among females with higher rates of gender inequality (20). 

Of the students, 2% reported regular drinking, which is far different from alcohol use rates reported in developed countries (70% in the US and 89% in Canada) (21). The rate is also much less than another study among university students in Tehran that reported the rate of 11.8% (22). Lower rates may be due to methodological differences, e.g., different definitions of regular alcohol user, survivorship bias as mentioned about smoking rates, or the real epidemiological difference due to religious and/or cultural issues. Although the prevalence of regular alcohol use is lower than other regions, the rate of binging is very high and almost all of the students who use alcohol had at least one episode of binging in the previous year, compared to 20% reported in other studies (23). One study found the alcohol availability as a risk factor for binging (24). In Iran, Liquor is not legally marketed, however, one may not conclude that they are not available. Liquor cannot be obtained legally, so people obtaining them on a regular basis may have a higher level of novelty seeking as a part of their characteristic, making them prone to binging. 

In the present study, no correlation was found between tobacco use and alcohol use/prescription drug misuse; however, other studies have reported a correlation (17, 25). Nevertheless, higher availability of drugs (as they are sold over the counter) and lower availability of alcohol may blur the correlation. 

In this study, the prevalence of overweight and obesity was 18.1% and 1.7%, respectively, which is not quite different from other studies (26). Five daily meals, including three main meals, are recommended as a healthy pattern of eating. Only half of the students had meals regularly, and this pattern is quite similar to that of previous studies (27). The majority of students had vegetables or fruits daily. Higher rates of vegetable and fruit intake might have been due to availability and relatively lower price of these products in Iran. Among students who had breakfast and other meals regularly, the rate of overweight was higher, which could be the result of inappropriate dieting and overeating. The rate of having meals outside home was not higher among those who were overweight or obese compared to those who had normal weight; this finding further confirms the bad eating habits of students. 

Despite what was expected, a negative correlation was found between being overweight or obese and having physical activity. Perhaps, for most of the students having regular physical activity was not regarded as a healthy behavior in their routine life style, but they only regard it as a mean to lose weight. 

Wearing a seatbelt as a driver or a front seat passenger was reported in two thirds of the students, which is comparable to previous reports (28). Exceeding speed limit was reported in the majority of students and was higher than the rates that had been previously reported in Iran, which might have been due to the age of the participants of this study. 

In terms of knowledge of screening for breast, cervical, and testicular cancer and commitment to do it, a huge gap was found between knowledge and behavior. Also, few other studies from Iran and other developing countries showed low rates of screening. Lack of education, either in the educational system and the public, and also the absence of family medicine system that can promote screening, may be the main obstacles of performing screening tests (29, 30). 

Many factors may affect positive and negative heath behaviors, including knowledge, beliefs and attitudes, legal constrains, social context, and economic status (13). Although lower health literacy leads to more negative health behaviors (31), there are discrepancies between health behaviors and knowledge (13). However, there is substantial evidence about effective interventions to promote health behaviors, especially web-based interventions (32, 33). Efficacy of these interventions and their long-term effects on individual and large-scale health status should be considered in future studies.

## Limitation

The present study had some limitations. First, the data were collected via self-reported questionnaires that could be the subject of some inaccuracies because of recall bias or social acceptability. The descriptive nature of the study limited the generalizability of the findings. Also, the study samples were university students; hence, the results should be very cautiously generalized to the general young population, as the young population who were not interested in healthy behaviors might have been underrepresented in this sample. Future studies should address the mentioned limitations.

## Conclusion

Overall, this study revealed that the majority of the students exhibited some health-promoting behaviors, including relatively healthy diet and physical activity. The behaviors that are more related to the health literacy, such as routine screenings, were less common. The proportion of students who did not avoid risky behaviors (e.g., smoking, unprotected sex, and binging) was relatively high, although this finding was not different from that of other countries. Thus, it is highly recommended that health promotion programs, with focus on disease prevention, health promotion, and reduction of risky behaviors, be implemented in universities. In addition, the students should be empowered with health literacy and behavior change techniques to choose healthy behaviors.
